# NADH dehydrogenase of *Trypanosoma brucei* is important for efficient acetate production in bloodstream forms

**DOI:** 10.1016/j.molbiopara.2016.10.001

**Published:** 2017-01

**Authors:** Sachin V. Surve, Bryan C. Jensen, Meredith Heestand, Muriel Mazet, Terry K. Smith, Frédéric Bringaud, Marilyn Parsons, Achim Schnaufer

**Affiliations:** a Center for Infectious Disease Research (Formerly Seattle Biomedical Research Institute), 307 Westlake Ave. N., Seattle, WA, 98109, USA; b Centre de Résonance Magnétique des Systèmes Biologiques (RMSB), UMR5536, Université de Bordeaux, CNRS, Bordeaux, France; c Biomedical Sciences Research Complex, University of St Andrews, North Haugh, St. Andrews KY16 9ST, United Kingdom; d Dept. of Global Health, University of Washington, Seattle, WA, 98195, USA; e Institute of Immunology & Infection Research and Centre of Immunity, Infection & Evolution, The University of Edinburgh, Edinburgh, EH9 3FL, United Kingdom

**Keywords:** *Trypanosoma brucei*, Mitochondrion, NDH2, Respiratory complex I, NADH:ubiquinone oxidoreductase, Acetate

## Abstract

In the slender bloodstream form, *Trypanosoma brucei* mitochondria are repressed for many functions. Multiple components of mitochondrial complex I, NADH:ubiquinone oxidoreductase, are expressed in this stage, but electron transfer through complex I is not essential. Here we investigate the role of the parasite’s second NADH:ubiquinone oxidoreductase, NDH2, which is composed of a single subunit that also localizes to the mitochondrion. While inducible knockdown of NDH2 had a modest growth effect in bloodstream forms, *NDH2* null mutants, as well as inducible knockdowns in a complex I deficient background, showed a greater reduction in growth. Altering the NAD^+^/NADH balance would affect numerous processes directly and indirectly, including acetate production. Indeed, loss of NDH2 led to reduced levels of acetate, which is required for several essential pathways in bloodstream form *T. brucei* and which may have contributed to the observed growth defect. In conclusion our study shows that NDH2 is important, but not essential, in proliferating bloodstream forms of *T. brucei*, arguing that the mitochondrial NAD^+^/NADH balance is important in this stage, even though the mitochondrion itself is not actively engaged in the generation of ATP.

## Highlights

• Various genetic mutants of NDH2 were created in bloodstream form *Trypanosoma brucei*.
• NDH2 null mutants showed a substantial reduction in growth.
• NDH2 ablation in a complex I deficient background led to severe growth restriction.
• Upon prolonged culture, parasites partially compensated for NDH2 deficiency.
• Loss of NDH2 led to reduced acetate, potentially contributing to the growth defect.

Respiratory chains of prokaryotes and eukaryotes are composed of complexes catalyzing oxidation of NADH along with translocation of protons across inner mitochondrial (in eukaryotes) or plasma (in prokaryotes) membranes resulting in gradient formation. This gradient in turn drives the ATP synthesis via ATP synthase (complex V). In addition to the five main complexes in mammalian mitochondria involved in energy production [1], some plants, fungi and parasites have additional enzymes that complement these complexes. Such enzymes include alternative oxidase [2], [3], [4] and alternative or Type II NADH dehydrogenases [5].

*Trypanosoma brucei* long slender bloodstream forms (BF) rely solely on glucose for their energy requirements; the glucose is metabolized primarily via glycolysis [6]. The mitochondria of these cells possess only two of the large complexes associated with the respiratory chain; complex I (NADH:ubiquinone oxidoreductase; cI), and complex V [7], [8], [9], [10]. Differentiation into the transmission competent, but non-proliferative short stumpy BF appears to be associated with up-regulation of cI [9], [10], [11]. Our previous characterization of cI subunits in *T. brucei* slender BF showed presence of multi-subunit complexes, but as to whether a complete cI is assembled remains unclear [12]. Nonetheless, successful deletion of two cI subunits (NUBM and NUKM) proved that electron transfer within cI is not essential in slender BF and that cI does not contribute significantly to NADH dehydrogenase activity in these cells [12]. These findings were surprising because mRNAs of the mitochondrially encoded subunits of cI are preferentially edited in BF to specify functional proteins. As at least two NAD^+^-dependent activities are known to be essential in BF (the glycine cleavage complex [13] and acetate production via pyruvate dehydrogenase or threonine dehydrogenase [14]), we reasoned that other enzymes in cI deficient lines either replace or complement cI’s NADH:ubiquinone oxidoreductase activity. The type II NADH dehydrogenase NDH2 appeared to be the most likely candidate, as the enzyme can transfer electrons from NADH to ubiquinone and was reported to be active in *T. brucei* − at least in the procyclic insect form (PF) [15], [16], [17]. Thus, NDH2 would be capable of regenerating sufficient NAD^+^ for use within the mitochondrion.

*T. brucei* NDH2 belongs to class A NDH2 enzymes, which are present in all three domains of life. The *T. brucei* enzyme, a single polypeptide of ∼54 kDa [16], utilizes a non-covalently attached FMN as a cofactor and was proposed to be the source of rotenone-insensitive NADH dehydrogenase activity in sucrose gradient fractions of PF lysates [15]. Using RNAi to target *NDH2* (Tb927.10.9440) in PF yielded slower growth and decreased mitochondrial membrane potential [17]. However, NADH:Q2 oxidoreductase activity did not change significantly in these *NDH2* knockdown cells [17]. The authors also proposed that the enzyme was facing the mitochondrial intermembrane space and not the matrix, contrary to the earlier publication [15]. Although presence of the NDH2 protein in slender BF was confirmed in recent proteomic studies [18], [19], its physiological role in BF is not known.

To further understand the role of NDH2 in slender BF, we generated and analyzed the effect of *NDH2* knockout (or conditional knockout, cKO) in wild type and cI deficient lines using *T. b. brucei* BF strain Lister 427. We first tested for essentiality by attempting to generate *NDH2* null parasites in the single-marker derivative of strain Lister 427 [20], using deletion constructs where the drug resistance genes were flanked by regions directly upstream and downstream of the *NDH2* coding sequence (see Supplemental Methods for details and primers). Deletion of *NDH2* in the resulting transfectants was confirmed by PCR (not shown) and genomic Southern analysis (Fig. S1A). In general, we observed a clear growth defect early after transfection, but parasites reproducibly were able to partially compensate to differing extents upon continued culture. For example, at 46 days in culture, the two knockout lines differed in their growth characteristics, with one showing slightly slowed growth and the other a much stronger decrease (Fig. S1B). Anecdotally, we noticed that both of these clones appeared to be sensitive to stress, such as recovery from frozen stocks.

**Fig. 1:**
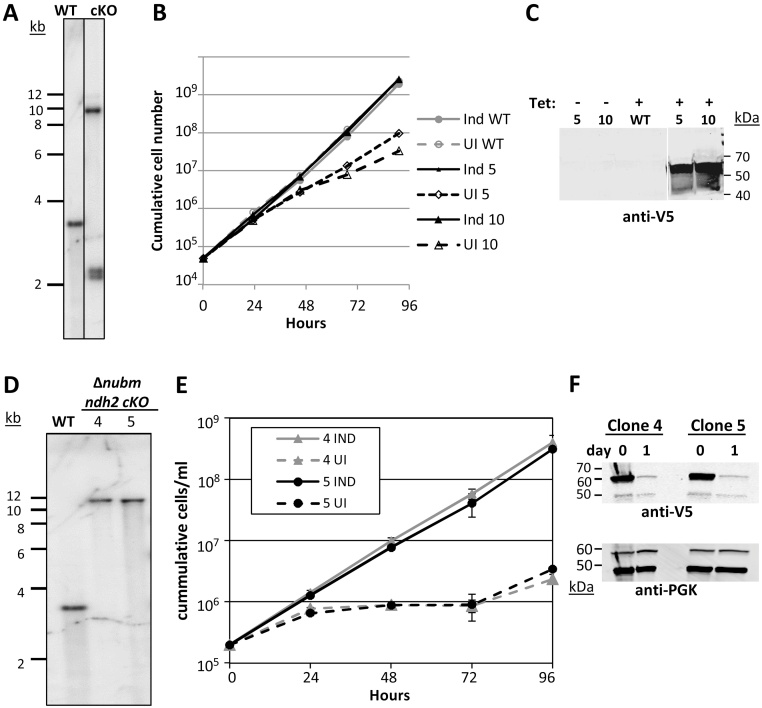
NDH2 is advantageous but not essential for growth of BF *T. brucei in vitro*. (A) Southern blot analysis of the *NDH2* locus. Genomic DNA was isolated from the parental ‘wild type’ (WT) line and *ndh2* cKO clone 10. Following digestion with SnaBI and probing with the *NDH2* CDS, the expected band sizes are: WT, 3.6 kbp; cKO, >5.5, 2.4, and 2.3 kbp. As the largest fragment in the cKO cell line represents integration of the plasmid bearing the complementing gene into one of the rDNA loci, it is not possible to accurately predict the fragment size. The lanes are from the same gel and hybridization. (B) Cumulative growth curves of tetracycline (Tet)-treated (induced, Ind) and uninduced (UI) *ndh2* cKO cells is compared to the parental WT. For uninduced (UI) cells Tet was removed from the medium at time point zero. Two clones (5 and 10) were analyzed. This experiment was performed in duplicate and 91% of the replicates on days 1–4 were within 15% of the mean. (C) Assessment by immunoblot of levels of ectopic, V5-tagged NDH2 in uninduced (-Tet) vs. induced cells (+Tet) on day 5. Gels (5 × 10^6^ cell equivalents per lane) were transferred to nitrocellulose membranes, blocked and incubated with mouse anti-V5 monoclonal antibody at 0.5 μg/ml. Anti-V5 was detected by goat anti-mouse IgG-IRDye 800CW using a Li-Cor Odyssey system. NDH2-V5 has a predicted molecular weight of ∼60 kDa. Lanes are from the same scan of the western blot. (D) Southern analysis confirming the *ndh2* cKO genotype in the Δ*nubm* background. Genomic DNA digested with SnaBI was analyzed by Southern blot using a probe consisting of the *NDH2* coding sequence. Expected sizes are: WT, 3.6 kbp; Δ*nubm ndh2* cKO clones 4 and 5, >5.6 kbp (the complementing copy is in one of the rDNA loci, so its exact size cannot be predicted). The *NUBM* knockout was confirmed in a previous study [12]. (E) Cumulative growth in the presence and absence of the inducer Tet of parasites with all endogenous alleles of *NUBM* and *NDH2* deleted and harboring an inducible, V5-tagged copy of *NDH2*. For uninduced (UI) cells Tet was removed from the medium at time point zero. Two individual clones, 4 and 5, were analyzed. Error bars mark the standard deviation of the triplicate data points. For both clones, the calculated doubling times were ∼9.3 h in the presence of Tet and >100 h in the absence of Tet (days 1–3). (F) Western analysis of NDH2-V5 expression in the cKOs upon Tet withdrawal, in parallel with panel E. The same blot was re-probed with anti-phosphoglycerate kinase (PGK) as a loading control. The return to normal growth rates 3–4 days after Tet removal is most likely due to loss of repression of the ectopic *NDH2* gene, as was seen in other experiments (see Fig. S2).

Given the clone-specific differences in the Δ*ndh2* parasites, and partial recovery of growth rates over time, we generated cKOs. The endogenous *NDH2* genes were deleted in parasites bearing a tetracycline (Tet)-regulated ectopic copy of *NDH2* (tagged with three V5 epitopes) (see Southern analysis, Fig. 1A). Removal of Tet was accompanied by slowed growth (the doubling time increased by approximately 1.5 fold), but the parasites continued to proliferate (Fig. 1B). Western analysis confirmed the knockdown of the ectopic protein (Fig. 1C). The C-terminal V5 tags did not interfere with function since induced cells showed growth rates similar to wild type (WT) cells. Thus, NDH2 appears to be beneficial, but not essential for *in vitro* growth of slender BF *T. brucei*.

In earlier work, we showed that slender BF parasites lacking cI subunits NUBM or NUKM,which are required for electron transfer within cI, have no growth defect *in vitro* or *in vivo* [12]. However, it is possible that normal levels of NDH2 are sufficient to fulfill cellular requirements for regeneration of NAD^+^ in the absence of cI. We therefore generated *ndh2* mutants in the previously characterized cI-deficient line, Δ*nubm* [12]. In several attempts, we obtained *ndh2* double knockouts only in the presence of an ectopic copy of *NDH2*. The *ndh2* cKOs in the Δ*nubm* parasites were confirmed by Southern blot analysis (Fig. 1D) and by PCR (not shown). A strong growth phenotype was observed upon NDH2 knockdown in parasites lacking cI function; after withdrawal of Tet, their growth slowed dramatically, nearly ceasing within 24 h (Fig. 1E). Growth began to recover three days to four days after Tet withdrawal, most likely due to loss of repression, as is common in *T. brucei* (Fig. S2). The growth phenotype of Δ*nubm ndh2* cKO cells was stronger than that seen for *ndh2* cKO clones (although NDH2 repression appeared to be even more stringent in the latter (compare Fig. 1C with Fig. 1F and Fig. S2), and additionally appeared to be stronger than the growth phenotype of the Δ*ndh2* clones. One possible explanation for our observation is that cI can partially compensate for NDH2 loss, which then would suggest that the two activities function in the same compartment, the mitochondrial matrix, consistent with an earlier report [15]. However, another study has suggested that NDH2 is localized to the intermembrane space of the mitochondrion in insect stage parasites [17].

**Fig. 2:**
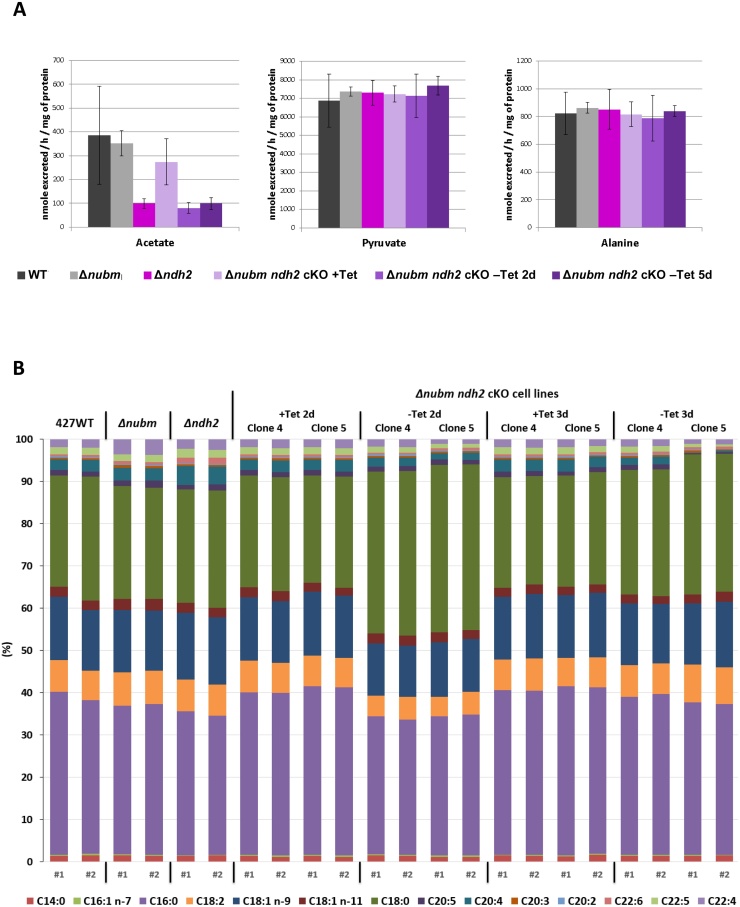
Metabolic changes after NDH2 ablation. (A) Ablation of NDH2 decreases the amount of acetate excreted by the parasites. The amount of acetate, pyruvate and alanine excreted by the WT cells and the Δ*nubm* and/or *ndh2* mutant cell lines (given as nmole/h/mg of protein) was determined by ^1^H NMR spectrometry as previously described [14]. Three biological replicates were performed for each cell line, except for WT (7 replicates) and Δ*nubm* (8 replicates). The standard deviations for each condition are indicated. Δ*ndh2* clone a3-10 and Δ*nubm ndh2* cKO clone 4 were used. (B) Characterisation and relative quantification of the total fatty acids present in the lipid extracts of duplicate biological replicates of each of the various clones. This was done by base hydrolysis and subsequent conversion of the fatty acids to their methyl esters allowing analysis by GC–MS as previously described [23].

To further probe the mechanisms by which loss of NDH2 function affects parasite metabolism, we considered various enzymes that would utilize NAD^+^ in slender BF. One candidate is the glycine cleavage complex. However, the excess thymidine in the standard growth medium would be expected to rescue any detrimental effects on that pathway [13]. Recently it was shown that mitochondrial production of acetate is essential in BF *T. brucei* and can proceed through two pathways that contribute roughly equally: one from pyruvate (the predominant end product of glycolysis) and the other from threonine, derived from the medium [14]. Enzymes required for these routes include two NAD^+^-dependent enzymes, pyruvate dehydrogenase and threonine 3-dehydrogenase, which were shown to be synthetically lethal (*i.e.* ablation of either gene alone was compatible with viability but simultaneous ablation of both genes causes death) [21]. We therefore examined by ^1^H NMR spectrometry the amounts of three relevant metabolites pyruvate, acetate and alanine (the two latter are minor products of pyruvate metabolism) excreted by WT, Δ*ndh2*, Δ*nubm*, and Δ*nubm ndh2* cKO parasites from glucose metabolism. No difference in pyruvate and alanine levels was observed between the various parasite lines (Fig. 2A). However, acetate showed a strong reduction in the *ndh2* null parasites and in the Δ*nubm ndh2* cKO line when NDH2 was not expressed (uninduced condition).

The reduced excretion of acetate raised the possibility that both cytosolic and mitochondrial fatty acid synthesis might be compromised after loss of NDH2 [22]. We therefore used gas-chromatography mass spectrometry (GC–MS) to quantitate relative changes in total cellular fatty acid content in the various cell lines after hydrolyzation of lipid extracts and conversion of the free fatty acids to the corresponding fatty acid methyl esters (FAME) [23]. For the Δ*nubm* cells the FAME analysis (Fig. 2B) indicated a relative increase in C22:4 fatty acids and a relative decrease in C16:0 fatty acids; according to our earlier study, this does not affect growth either *in vitro* or *in vivo* [12]. The most obvious changes in Δ*ndh2* parasites were a relative increase in C20:4 fatty acids and, similar to Δ*nubm* cells, a relative decrease in C16:0 fatty acids. NDH2 knockdown in the Δ*nubm* background also resulted in a reproducible, but for the most part temporary, shift in fatty acid composition that was remarkably similar for clones 4 and 5. Forty-eight hours after washing away Tet, C18:0 was relatively increased at the expense of most other fatty acids. After 72 h fatty acid content was largely normal again for both clones, although clone 5 continued to show a substantial relative reduction for some fatty acids, in particular C20:4.

These findings raised the question as to why the uninduced Δ*nubm ndh2* cKO parasites showed a more severe growth defect and shift in fatty acid content compared to Δ*ndh2* cells, despite a similar reduction in the amount of acetate excreted. There are several potential explanations for these observations. It is possible that the Δ*ndh2* parasites produced more acetate than uninduced Δ*nubm ndh2* cKO cells, allowing for more *de novo* fatty acid biosynthesis and/or elongation and thus faster growth rates (for technical reasons we could only measure excretion, not production). However, this explanation seems unlikely given that *T. brucei* requires only a very minor fraction of the acetate it produces for lipid biosynthesis (∼4% in procyclic forms [14]). Alternatively, some re-expression of NDH2 might have occurred in the Δ*nubm ndh2* cKO cells due to partial loss of repression at the time of the experiment (see, for example, Fig. S2). Finally, the growth phenotype and perturbed fatty acid content may not have been primarily due to acetate depletion but due to other metabolic pathways that had been affected by impaired NAD^+^ regeneration. For example, ablation of the enzyme succinyl-CoA synthetase, activity of which depends on acetyl-CoA production and thus secondarily on NAD^+^ regeneration, was reported to result in the rapid death of BF *T. brucei* [24].

Taken together, our data shows that NDH2 is an important, but not essential, factor in maintaining the mitochondrial redox balance in slender BF *T. brucei*. The temporary cessation of growth of the conditional knockdowns in a genetic background lacking cI function, and our inability to obtain mutants that were genetically null for both activities, could indicate synthetic lethality of the two NADH:ubiquinone oxidoreductases. This requires further investigation, perhaps with the help of alternative genetic tools such as Cre-lox [25] or CRISPR/Cas9 [26], [27], [28].
