# Supplementary material for: NADH dehydrogenase of Trypanosoma brucei is important for efficient acetate production in bloodstream forms
Source: Mol Biochem Parasitol. 2017 Jan;211:57–61. doi: 10.1016/j.molbiopara.2016.10.001 (PMC5225879; doi:10.1016/j.molbiopara.2016.10.001)
Supplement: Supplementary file 1 [file mmc1.pdf]

Supplementary Material

Supplemental Methods

Transfection and Growth assays. T. brucei bloodstream forms (BF) were grown in HMI-9 medium with 10% heat-inactivated fetal bovine serum at 37°C and 5% CO_2. Drugs to maintain selection for transgenes were included when relevant: hygromycin (1 μg/ml), puromycin (0.1 μg/ml), blasticidin S (3 μg/ml) and phleomycin (1 μg/ml). BF were transfected using Amaxa Nucleofector program X-001 [1,2]. Briefly, $3X10^7$ log phase BF cells were harvested by centrifugation and pellets were resuspended in 100 μl of Human T-cell Nucleofector solution plus 2-3 μg of DNA construct. Transfected cells were diluted 1:10 and 1:100 and incubated at 37°C for at least 6 h before adding appropriate drugs as described above. Protein expression from tetracycline (Tet)-regulated constructs was induced with 1 to 2 μg/ml of Tet. *In vitro* and *in vivo* growth analysis was carried as described [3].

Conditional ndh2 *knockouts in Lister 427 BF*. Primers are listed in Table S1. Deletion constructs were generated using plasmids pLew13 and pLew90 [4]. The regions targeting the drug resistance cassette were 500 bp region upstream of the *NDH2* coding sequence (CDS), Tb927.10.9440 (amplified using PCR primers 1 and 2 with NotI and MluI sites, respectively) and the last 456 bp from the 3' end of the *NDH2* CDS (amplified using primers 3 and 4, with XbaI and StuI sites, respectively). These regions were used to flank T7 RNA polymerase and *NEO* genes (first allele knockout) and the Tet repressor and *HYG* gene cassette (second allele knockout), as described [5]. For ectopic expression, V5-tagged NDH2 was generated by cloning the *NDH2* CDS into the pLew-3V5-PAC plasmid [3] via HindIII and BamHI sites (primers 17 and 18). Constructs were confirmed by restriction analysis and sequencing, and were digested with NotI before transfection.

Null mutants in single marker BF and ndh2 *cKOs in Δ*nubm *BF*. A stitching PCR approach was employed to generate deletion constructs [6]. The constructs were designed such that drug resistance genes were flanked by regions upstream and the last 456 bp of the *NDH2* CDS. First, these regions were amplified from genomic DNA using Phusion polymerase system (Thermo) with an overhang (20 bp) of the sequence from drug resistance genes (*HYG*, *PHLEO* or *PAC*) (primers 5-10). CDSs for the drug resistance genes were also amplified using Phusion PCR system (primers 13-16). The first round amplicons were then stitched together in by amplification using primers situated at 5' end of the 5' UTR and 3' end of the 3'UTR (primers 11 and 12). The second round amplicons were then dA-tailed using GoTaq Polymerase (Promega), and cloned into pGEM-T Easy (Promega) vector. An ectopic copy of V5 tagged NDH2 was generated by cloning the *NDH2* CDS into pLew79-3V5-BSD, which was constructed by swapping the *PAC* gene from pLew79-NDH2-V5-PAC plasmid with the *BSD* gene from pLew100-V5-BSD (kind gift from George Cross) using SnaBI sites.

Initial confirmation of gene disruptions was done by PCR. Subsequent Southern analysis confirmation was performed as shown in the main text.

Table S1. Primers

Primer	Name	Sequence (5'-3')	Use
		Primers for *NDH2* cKO	
1	Ndh 5F	ATGAGCGGCCGCATTTTATGTGTGAAGGGGAGG	Amplify 5' UTR
2	Ndh 5R	ATGAACGCGTCTCGAGACTTGTCGTTCAGATCTCCACT	Amplify 5' UTR
3	Ndh 3F	ATATCTAGAATTTAAATCTGCTGTCGCTTCTCGTCA	Amplify 3' CDS end
4	Ndh 3R	ATAAGGCCTGCGGCCGCCTACTCGTTCTGTTTCTTTG	Amplify 3' CDS end
		Primers for stitching KOs	
5	Ndh 5F-642	AGATATTGTCCGTACTGCCTC	fusion 5' UTR
6	NDH 5R Phleo	**GAACGGCACTGGTCAACTTGGCCAT**CTTCCTTCTTTTTCCTCCCCTC	Fusion 5' UTR to *PHLEO*
7	NDH 5R PAC	**GTGGGCTTGTACTCGGTAACCAT**CTTCCTTCTTTTTCCTCCCCTC	Fusion 5'UTR to *PAC*
8	NDH 3F Phleo	**GTGGCCGAGGAGCAGGACTGA**GTGAAGTTTGTGTACCAAGCAAC	Fusion 3' UTR to *PHLEO*
9	NDH 3F PAC	**GACCCGCAAGCCCGGTGCCTGA**GTGAAGTTTGTGTACCAAGCAAC	Fusion 3' UTR to *PAC*
10	Ndh 3R+931	AGAGGAGGCAAAAAGGTTCA	Fusion 3' UTR
11	Ndh 5F-566	TGTGTGGAAAGGGAACGAAG	Final fusion PCR
12	Ndh 3R+856	GAGAGTGCGCCAGTGTCATAC	Final fusion PCR
13	Phleo F ATG	ATGGCCAAGTTGACCAGTGCCGTTC	Amplify resistance gene
14	Phleo R Stop	TCAGTCCTGCTCCTCGGCCAC	Amplify resistance gene
15	PAC F ATG	ATGGTTACCGAGTACAAGCCCAC	Amplify resistance gene
16	PAC R Stop	TCAGGCACCGGGCTTGCGGGTC	Amplify resistance gene
		Primers for complementation	
17	Ndh2 Hind F	ATGCAAGCTTATGATATGCCGCACATCTT	Cloning Gene
18	Ndh2 Bam R	GCATGGATCCCTCGTTCTGTTTCTTTGTCG	Cloning Gene

Restriction sites added for cloning are underlined. Bases in bold anneal with the drug resistance genes in the fusion PCR, while standard font matches *NDH2* flanking sequence. In the primer names, negative numbers indicate nt upstream of *NDH2* start codon (forward) and positive number indicate position downstream of *NDH2* stop codon (reverse primers).

References

[1] Burkard G, Fragoso CM, Roditi I. (2007) Highly efficient stable transformation of bloodstream forms of *Trypanosoma brucei*. *Mol Biochem Parasitol* 153: 220-3.

[2] Cross GAM. Tools for genetic analysis in *Trypanosoma brucei*. Available from URL: http://tryps.rockefeller.edu/trypsru2_protocols_index.html

[3] Surve S, Heestand M, Panicucci B, Schnaufer A, Parsons M. (2011) Enigmatic presence of mitochondrial complex I in *Trypanosoma brucei* bloodstream forms. *Eukaryot Cell* 11: 183-93.

[4] Wirtz E, Leal S, Ochatt C, Cross GA. (1999) A tightly regulated inducible expression system for conditional gene knock-outs and dominant-negative genetics in *Trypanosoma brucei*. *Mol Biochem Parasitol* 99: 89-101.

[5] Ochatt CM, Butikofer P, Navarro M, Wirtz E, Boschung M, Armah D, *et al.* (1999) Conditional expression of glycosylphosphatidylinositol phospholipase C in *Trypanosoma brucei*. *Mol Biochem Parasitol* 103: 35-48.

[6] Merritt C, Stuart K. (2013) Identification of essential and non-essential protein kinases by a fusion PCR method for efficient production of transgenic *Trypanosoma brucei*. *Mol Biochem Parasitol* 190: 44-9.

Supplementary Figures

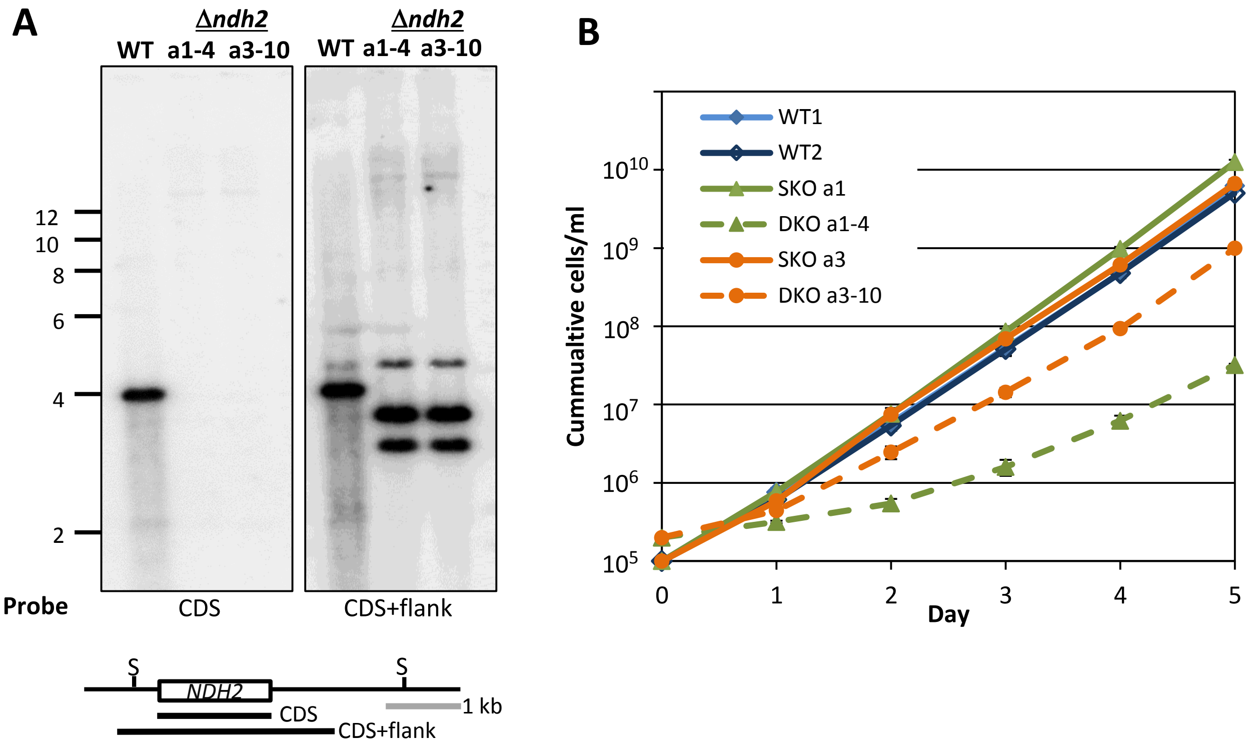

Fig. S1. Generation and growth analysis of Δ*ndh2* cell lines. (A) Genomic Southern blot of Δ*ndh2* clones. Genomic DNA was digested with SnaBI (S), fractionated by agarose gel electrophoresis, transferred to a membrane and probed with a fragment consisting of either the coding sequence (CDS) alone or the CDS plus 5' and 3' flanking regions (CDS+flank) as shown below. Expected sizes for the CDS probe are: WT, 3.6 kbp; Δ*ndh2* clones a1-4 and a3-10, no band. For the 'CDS+flank' probe expected sizes are: WT, 3.6 and 4.1 kbp; Δ*ndh2* clones, 2.5, 2.7, and 4.1 kbp. (B) Growth curve of Δ*ndh2* (DKO) mutants compared to their parental single knockout clones (SKO) and the parental single marker line (WT). The curve shows the cumulative cell concentration per ml with error bars marking the standard deviation of the triplicate data points. This experiment was conducted at 46 days of culture post-transfection.

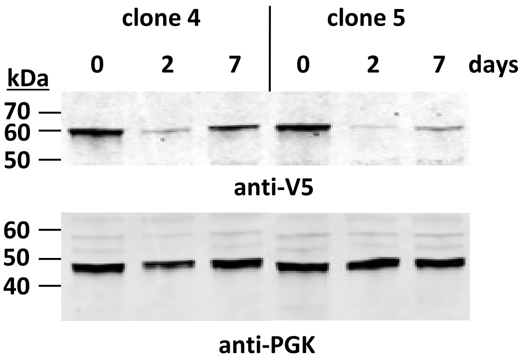

Fig. S2. Re-expression of NDH2-V5 in the Δ*nubm ndh2* cKOs several days after Tet withdrawal. Western blot analysis showing that after withdrawal of Tet, NDH2-V5 expression had strongly decreased by day 2 as compared to day 0, but increased again at later time points. The same blot was re-probed with anti-PGK as loading control. The samples analyzed here were derived from a repeat of the growth experiment shown in Fig. 1E. Proliferation slowed from day 1 to day 3 and then recovered to near normal rates by day 5.
